# Short-chain fatty acids (SCFAs) as potential resuscitation factors that promote the isolation and culture of uncultured bacteria in marine sediments

**DOI:** 10.1007/s42995-023-00187-w

**Published:** 2023-07-29

**Authors:** Chun-Shui Sun, Liu-Yan Zhou, Qi-Yun Liang, Xiao-Man Wang, Yi-Xuan Lei, Zhen-Xing Xu, Feng-Qing Wang, Guan-Jun Chen, Zong-Jun Du, Da-Shuai Mu

**Affiliations:** 1 Marine College, Shandong University, Weihai, 264209 China; 2 State Key Laboratory of Microbial Technology, Institute of Microbial Technology, Shandong University, Qingdao, 266237 China; 3 Weihai Research Institute of Industrial Technology of Shandong University, Weihai, 264209 China; 4 Institute of Microbiology Applications, Xinjiang Academy of Agricultural Sciences, Urumqi, 830000 China; 5 Tancheng County Inspection and Testing Center, Tancheng, 276100 China; 6 Department of Applied Biological Chemistry, Graduate School of Agricultural and Life Sciences, The University of Tokyo, Bunkyo-Ku, Tokyo, 113-8657 Japan; 7 Max Planck Institute for Marine Microbiology, Celsiusstraße 1, 28359 Bremen, Germany

**Keywords:** Marine bacteria, SCFAs, VBNC, Resuscitation culture

## Abstract

Many marine bacteria are difficult to culture because they are dormant, rare or found in low-abundances. Enrichment culturing has been widely tested as an important strategy to isolate rare or dormant microbes. However, many more mechanisms remain uncertain. Here, based on 16S rRNA gene high-throughput sequencing and metabolomics technology, it was found that the short-chain fatty acids (SCFAs) in metabolites were significantly correlated with uncultured bacterial groups during enrichment cultures. A pure culture analysis showed that the addition of SCFAs to media also resulted in high efficiency for the isolation of uncultured strains from marine sediments. As a result, 238 strains belonging to 10 phyla, 26 families and 82 species were successfully isolated. Some uncultured rare taxa within *Chlorobi* and *Kiritimatiellaeota* were successfully cultured*.* Amongst the newly isolated uncultured microbes, most genomes, e.g. bacteria, possess SCFA oxidative degradation genes, and these features might aid these microbes in better adapting to the culture media. A further resuscitation analysis of a viable but non-culturable (VBNC) *Marinilabiliales* strain verified that the addition of SCFAs could break the dormancy of *Marinilabiliales* in 5 days, and the growth curve test showed that the SCFAs could shorten the lag phase and increase the growth rate. Overall, this study provides new insights into SCFAs, which were first studied as resuscitation factors in uncultured marine bacteria. Thus, this study can help improve the utilisation and excavation of marine microbial resources, especially for the most-wanted or key players.

**Supplementary Information:**

The online version contains supplementary material available at 10.1007/s42995-023-00187-w.

## Introduction

Bacteria, which are ubiquitous in the marine environment, provide a valuable resource that is still rarely explored or utilised. Through molecular methods, especially 16S rRNA gene analysis, valuable information that is independent of culturing can be provided. However, it is only through cultures that the physiological and ecological properties of these resources can be fully characterised (Wang et al. 2021). Many factors contribute to the discovery of microorganisms that are not yet cultured with dormancy being considered one of the most difficult factors to overcome when attempting to isolate and culture most marine bacteria (Zhang et al. 2021). Although a “simulated habitat” strategy has successfully resulted in the culture of some uncultured microbial groups (Jung et al. 2022), it is not effective on dormant bacteria or those found in low abundance.

In unfavourable environmental conditions, such as nutrient limitation, desiccation, extreme temperatures or host responses, some bacteria persist in a metabolically inactive state but remain viable but non-culturable (VBNC) (Oliver 2010), i.e. they are temporarily unable to produce colony-forming units by plating on standard nutrient agar (Kaprelyants and Kell 1993). In addition, a sudden transition from low concentrations on environmental substrates to high concentrations on standard microbial media would decrease the viability of oligotrophs, leading to incubation failure or induction of VBNC (Colwell 2000). Such non-culturable cells need resuscitation procedures for them to be converted into a metabolically active state*.* Despite the progress that has been made in research on possible reactivation mechanisms, little is known about the biochemical basis of these processes (Nikitushkin et al. 2013). As a result, our understanding of marine microbial ecology remains limited. Resuscitation of dormant microorganisms under laboratory conditions is the key to cultivating uncultured marine bacteria (Mu et al. 2021).

Scientists have successively identified a variety of resuscitation factors, including physical stimuli, chemical stimuli, active proteins or host-associated stimuli (Zhang et al. 2020). Resuscitation of *Vibrio cholerae*, which enters the VBNC state to survive cold stress, can be induced either by a temperature upshift or addition of an anti-dormancy stimulant such as resuscitation promoting factors (Rpfs) at an appropriate temperature (Debnath and Miyoshi 2021). Additionally, Rpfs can be used as additives to enhance the enrichment and isolation of microbial communities (Li et al. 2014; Su et al. 2023). *Escherichia coli* spp. that are induced into the VBNC state can also be resuscitated by the addition of a combination of methionine, glutamine, threonine, serine and asparagine (Pinto et al. 2011). For *Salmonella enteritidis*, pyruvate endows VBNC cells with the ability to synthesise macromolecules, such as DNA and proteins, which are then resuscitated to the growing and colony-forming state during the resuscitation process (Morishige et al. 2013). Senoh et al. (2012) found that low temperature-induced VBNC cells of different enteric pathogenic species could be converted to the culturable state by co-culture with several eukaryotic cell lines. Most of the reported resuscitation factors focussed on pure cultures in the laboratory and on model or indicator strains (Zhang et al. 2020). A class of resuscitation factors that can promote the recovery of certain classes of microorganisms in natural environments, especially in marine environments is urgently needed.

In our previous study, through a meta-transcriptomics analysis, it was found that 80% of marine bacteria that were isolated and cultured using enrichment cultures, showed shifts in metabolic levels from low to high, implying changes from dormant to active states (Mu et al. 2018). It was assumed that intermediate metabolites of organic matter degradation were produced during the promoted the recovery of some uncultured bacteria. It has been reported (Lennon and Jones 2011) that through a complex sequence of biotic mineralization processes, degradation of organic matter is always accompanied by a succession of microbial communities.

During the anaerobic degradation of organic matter, SCFAs are key intermediates arising from primary fermentation and are utilised as substrates by bacteria, such as sulphate-reducing bacteria and methanogens, during secondary fermentation in marine sediments. SCFAs in sediments are controlled by bacterial activity and affect the composition of microbial communities. In marine sediments, the concentrations of SCFAs determine the microbial distributions in different sediment layers, suggesting their influence on microbial growth and microbial community composition (Glombitza et al. 2019). However, whether or how SCFAs affect microbial community viability remains unclear.

Here, an attempt is made to understand potential resuscitation factors that increase the isolation of uncultured bacteria during enrichment culturing. Through 16S rRNA gene high-throughput sequencing and targeted metabonomic technologies, the association of intermediate metabolites with the succession of microbial communities in five enrichment periods (e.g. D00, D05, D12, D21 and D30) were studied and an attempt was made to isolate uncultured marine bacteria by simulating the composition of the enrichment medium. To study the effect of SCFAs on VBNC bacteria, a strain of *Marinilabiliales* was introduced into the VBNC state and resuscitated by adding SCFAs to the enrichment medium. Additionally, growth curve tests were performed to determine the effects of SCFAs on the growth of stress-induced bacteria. This study offers new insights into the effects of SCFAs and is the first to verify the recovery factors in marine bacteria. It puts forward a new approach to promote the isolation of marine bacteria, especially for rare or key players.

## Results and discussion

### Dynamics of bacterial community diversity during enrichment culturing

**Fig. 1:**
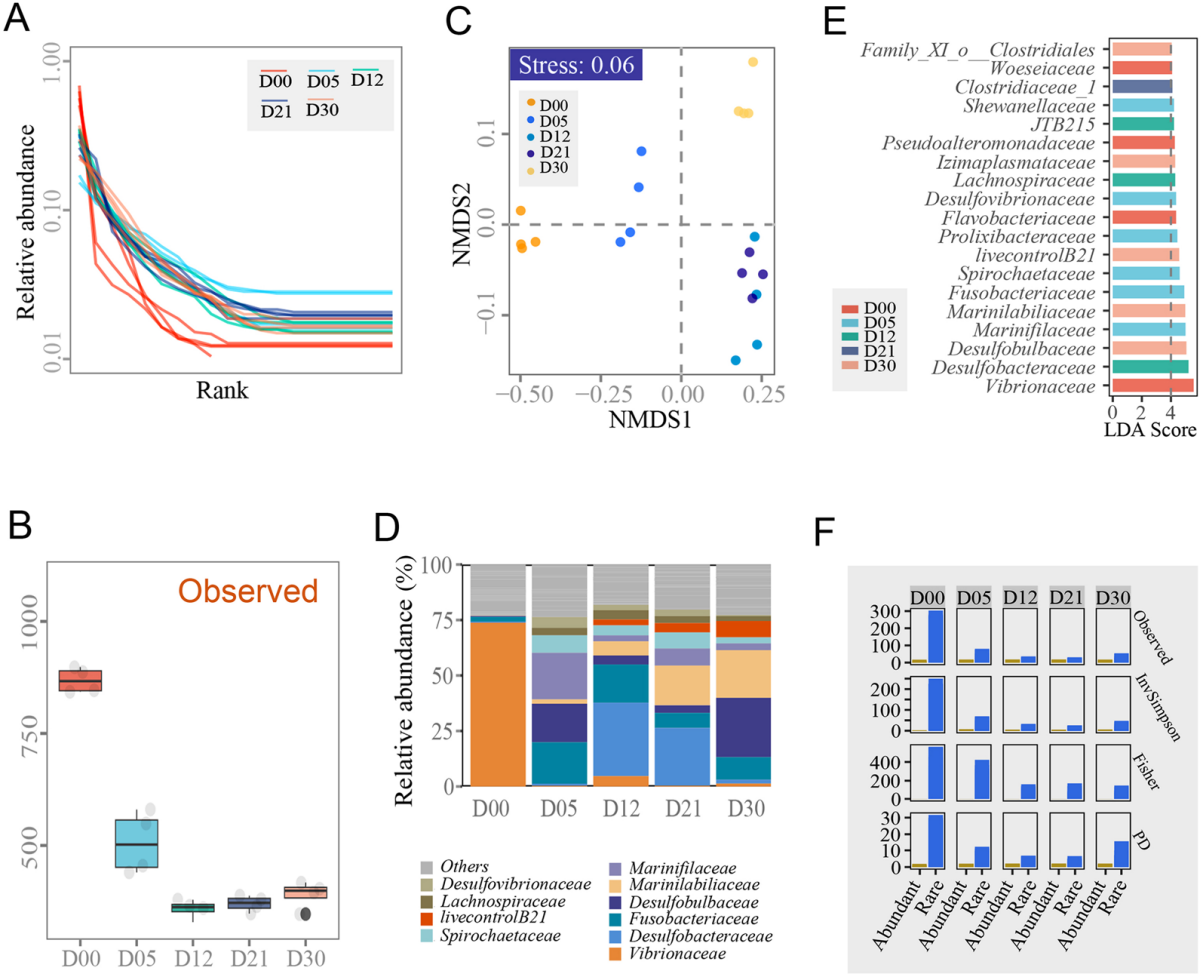
Analysis of community compositions and structures. **A** Rank abundance distributions of all incubation stages. The different incubation stages were randomly coloured. **B** Summary of OTUs detected in all incubation stages. **C** NMDS analysis of community structures and functions. **D** The abundant families in all incubation stages. The different abundant families are randomly coloured. **E** The discriminative abundant families in all incubation stages on the basis of LEfSe analysis. The different incubation stages were randomly coloured. D00, D05, D12, D21 and D30 represent incubation Days 0, 5, 12, 21 and 30, respectively, in all panels. **F** The alpha diversities of the abundant and rare taxa communities in all incubation stages. D00, D05, D12, D21 and D30 represent incubation Days 0, 5, 12, 21 and 30, respectively

A total of 700,820 high-quality sequences were obtained after quality control of high-throughput sequencing the 16S rRNA genes (the V3–V4 region). Based on a 97% cutoff, a total of 1601 OTUs were identified in the 20 samples, ranging in number from 329 to 898. The communities in the incubation stages (e.g. D05, D12, D21 and D30) had a more even distribution than those in raw marine sediments (Fig. 1A). The number of observed OTUs decreased significantly after incubation and remained stable from Days 5 to 30 (Fig. 1B). The structure and the functional redundancy indices of the bacterial communities were significantly different amongst all incubation stages (Fig. 1C, Supplementary Table S1). The alpha diversities of the abundant taxa communities in all incubation stages did not show significant differences, whilst the alpha diversities in the rare taxa communities showed significant differences amongst different incubation stages. Furthermore, the alpha diversities of the abundant taxa communities were smaller than those of the rare taxa communities in all incubation stages (Fig. 1F).

To compare the compositional changes in microbial communities in different enrichment culture periods, the microbial communities before the enrichment culturing (D00) were investigated first and these detected a large number of phyla. *Proteobacteria* (average 85.67%) was the most abundant phylum, followed by *Bacteroidetes* (average 6.47%),* Fusobacteria* (average 2.15%), *Acidobacteria* (average 1.11%), *Actinobacteria* (average 0.93%), *Planctomycetes* (average 0.84%) and *Firmicutes* (average 0.84%) (Supplementary Table S2). At the family level, before enrichment culturing, *Vibrionaceae* (average 73.74%) was the most abundant family, followed by *Flavobacteriaceae* (average 4.89%), *Pseudoalteromonadaceae* (average 3.72%), *Woeseiaceae* (average 2.41%) and *Fusobacteriaceae* (average 2.15%) (Fig. 1D–E) (Supplementary Table S3).

In different enrichment periods, the microbial community compositions showed significant changes. Compared with D00, the dominant phyla became *Bacteroidetes* (average 29.73%), *Proteobacteria* (average 28.90%), *Fusobacteria* (average 18.7%) and *Firmicutes* (average 12.78%) in D05, D12, D21 and D30, respectively, which had the same dominant phyla as D05, whilst *Spirochaetes*, *Tenericutes* and *Synergistetes* increased in abundance significantly compared to D05. The phyla *Proteobacteria*,* Actinobacteria*,* Acidobacteria* and *Planctomycetes* decreased by different degrees compared with D00.

The dominant families showed few differences at different enrichment stages. *Marinifilaceae* (average 20.85%), *Fusobacteriaceae* (average 18.17%), *Desulfobulbaceae* (average 17.34%) and *Spirochaetaceae* (average 7.99%) accounted for approximately 65% of the total bacterial population of D05. In this period, which is known as the acidifying period (Mu et al. 2018), a series of acid-producing bacteria increased significantly in abundance, although they were not the dominant group, for example, *Clostridiaceae_1* (average 2.67%), *Family_XI_o__Clostridiales* (average 2.17%), and *Clostridiaceae_4* (average 1.61%).

In D12, *Desulfobacteraceae* (average 33.14%) increased significantly and become the most abundant family. *Marinilabiliaceae* (average 6.46%) and *Clostridiales Incertae Sedis* (average 1.47%) showed significant increases whilst the average abundance of *Desulfobulbaceae* (average 4.03%) and *Marinifilaceae* (average 2.65%) decreased significantly compared with D05. The D21 period possessed a similar dominant family structure as D12, whilst the average abundances fluctuated to different degrees.

For the D30 period, *Desulfobulbaceae* (average 26.71%) increased to become the most abundant family. The abundance of *Desulfobacteraceae* (average 1.77%) decreased sharply from 25.97% in D21. *Synergistaceae* (average 1.45%), *norank_o__Clostridiales* (average 1.43%) and *norank_c__Latescibacteria* (average 1.41%) had lower abundances (less than 0.5%) in D00, D05, D12 and D21 but increased significantly in the last period of enrichment culturing. There were 3 to 5 abundant families (relative abundances > 5%) in the incubation stages (e.g. D05, D12, D21 and D30), whilst there was only one abundant family (the family *Vibrionaceae*, relative abundance > 5%) in the incubation stage (D00) (Fig. 1E). In detail, the families *Fusobacteriaceae* and *Marinilabiliaceae* were the most abundant taxa in the incubation stages from Days 5 to 30 and Days 12 to 30, respectively. Based on the LEfSe results, there were more than 3 significantly different and abundant families in all incubation stages, except in the incubation stage at Day 21 (Fig. 1E). These results were consistent with the findings of Mu et al. (2018).

During the enrichment culturing, the aerobic bacteria in the culture medium (e.g. *Flavobacteriaceae*, *Pseudoalteromonadaceae* and *Woeseiaceae*) were extensively suppressed, whilst anaerobic bacteria (e.g. *Clostridiaceae_1*, *Desulfobacteraceae* and *Fusobacteriaceae*) and facultative anaerobes (e.g. *Marinilabiliaceae* and *Marinifilaceae*) became the dominant groups. In the late stage of the enrichment culture, the strictly anaerobic bacteria, *Spirochaetaceae* and *Synergistaceae*, showed increasing relative abundances. This can be explained because the exhausted oxygen may be an important reason for the changes in microbial community structure.

The nutrient concentrations in the culture medium and environmental factors also changed during the enrichment stages, which is another reason for the changes in the microbial community structures. As the enrichment culture proceeded, the nutrient concentrations in the medium varied from the initial nutrient components (e.g. yeast powder and protein) to partially added nutrient components and microbial extracellular metabolites. The added proteins and lipids were actively utilised by bacteria within 5 days of the commencement of the enrichment culture. Along with the degradation of macromolecular organic matter, physical and chemical characteristics were also altered. The anaerobic organic carbon degradation processes include hydrolysis, fermentation and anaerobic respiration. It was notable that sulphate-reducing bacteria became a dominant group of bacteria during the enrichment culture. Sulphate-reducing bacteria (SRM) are essential oxidizers of fermentation products in marine sediments (Liang et al. 2023). The degradation of both proteins and lipids induces a rapid accumulation of several SCFAs.

Here, it was assumed that in addition to providing the necessary nutrients to facilitate bacterial growth, the extracellular metabolites produced by the bacteria during the enrichment culture may also have promoted the recovery of some bacterial groups, bringing them from dormancy to an active metabolic state.

### Correlations of physical and chemical factors and extracellular metabolites with bacteria during the enrichment culture

To examine the relationships between physical and chemical factors, intermediate metabolites, SCFAs and uncultured bacteria during the enrichment culture, changes in physical and chemical factors, and intermediate metabolites and SCFAs were measured during different enrichment culture periods, and a Mantel test was used to analyse their correlations with microbial communities.

### Physical and chemical factors

The physical and the chemical factors of the medium during the enrichment culture were determined with the analysis showing that Ca^2+^, Mg^2+^, Na^+^, K^+^, Cl^−^, NH_4_^+^ and Br^−^ concentrations declined from the D00 to D21 stages, although there was an inflexion point at the D21 stage, after which they slightly increased. The DOC and SO_4_^2−^ concentrations showed similar a similar pattern of change, first decreasing rapidly and then stabilising. DIC, however, showed the opposite trend, increasing significantly at first and then stabilising.

**Fig. 2:**
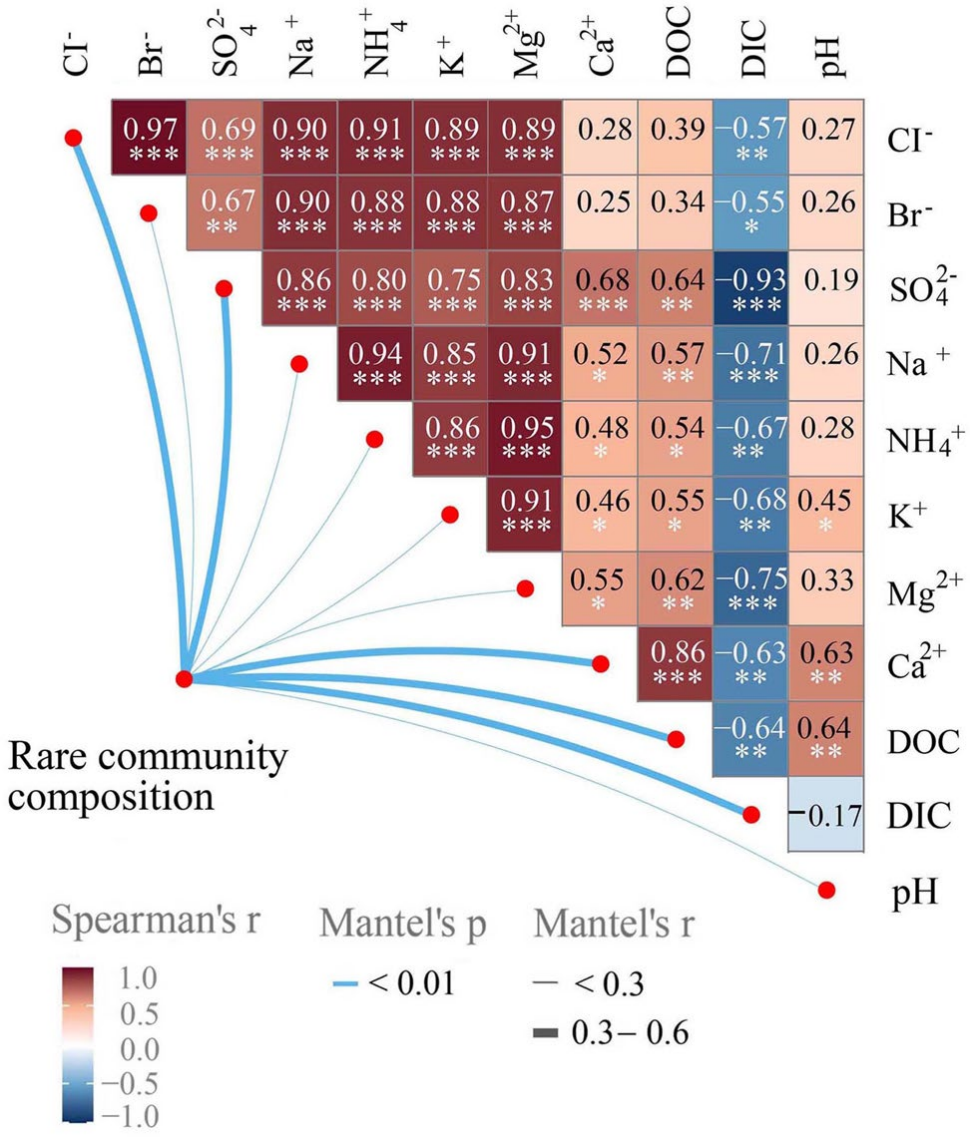
Correlations amongst physical and chemical factors. Mantel test results between rare community compositions and different physical and chemical factors and the Spearman correlation analysis amongst all physical and chemical factors

Strong correlations were found amongst the rare communities and SO_4_^2−^, DOC and DIC (Fig. 2), suggesting that rare communities might be crucial participants in the sulphur and carbon cycles. In addition, it was found that the pH of the medium decreased from D00 to D21. It was speculated that a certain number of organic acids, such as acetic acid, propionic acid, succinic acid and citric acid, were produced by specific anaerobic bacteria through anaerobic fermentation of indigestible carbohydrates.

### Extracellular metabolites

Together with the decomposition of macromolecular organic matter, a large number of intermediate metabolites were released into the enrichment culture. A GC–MS/LC–MS was used to analyse the extracellular metabolite composition, named P200, in the enrichment culture, in an attempt to find the key metabolites that were significantly related to the uncultured bacterial groups. P200 refers to the nearly 200 metabolites that are significant in life-related basal metabolism through 32 KEGG pathways.

A total of 36 P200 metabolites were detected. The concentrations of 1-methylxanthine, vitamin B6, imidazole and kynurenic acid and tryptamine contents increased significantly at the D05 stage but then tended to decline over time. In addition, creatinine, dihydrothymine, 4-pyridoxic acid, 5′-deoxyadenosine, 5-aminopentanoic acid and 5-amino-4-carbamoylimidazole (AICA) concentrations also increased in different enrichment periods.

There was an interdependence between community structures and metabolites, and the changes in metabolite concentrations were not only affected by changes in the microbial communities but also with specific microbial groups.

**Fig. 3:**
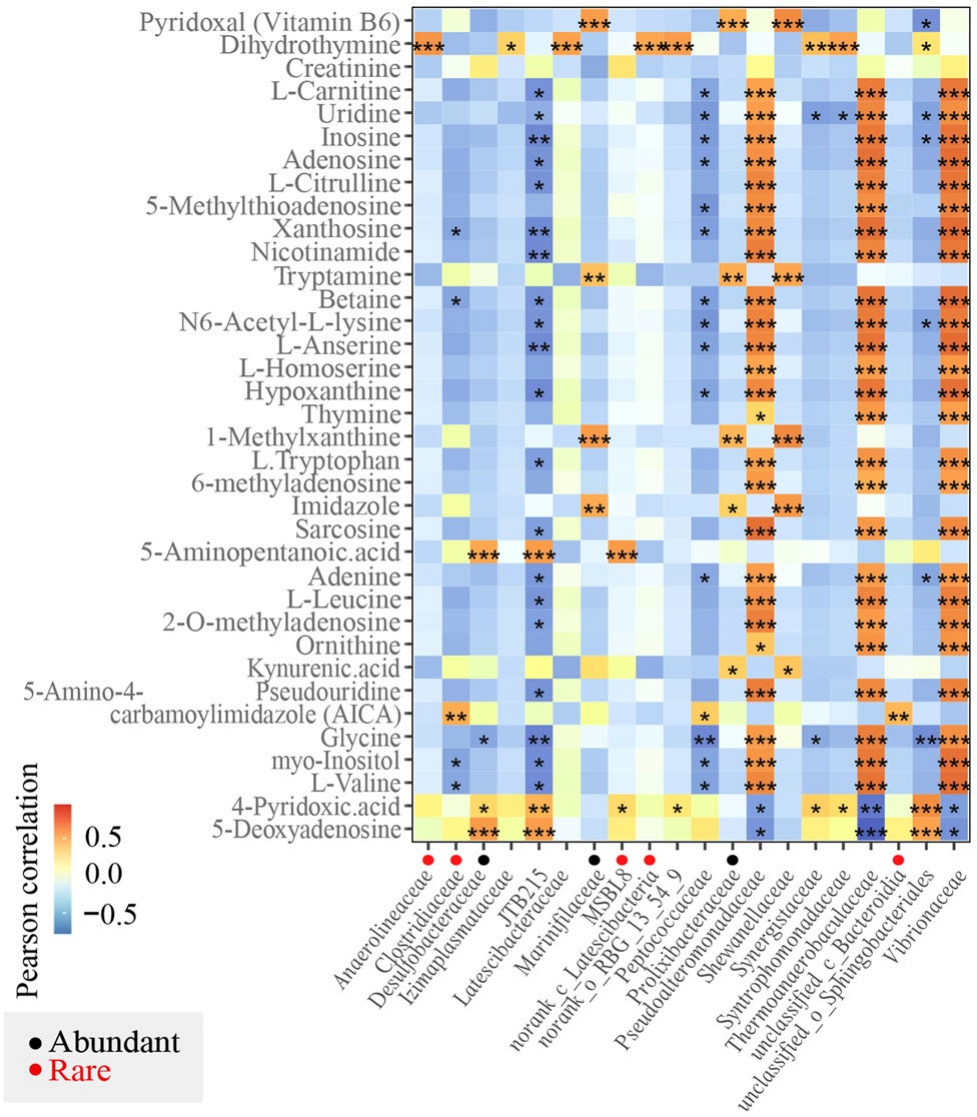
Correlations amongst community compositions and metabolites. Pearson correlations amongst different families in all incubation stages and representative metabolites. The gradient colours represent the different correlations. The black points are within the abundant taxa communities, and the red points are within the rare taxa communities in all incubation stages. *0.01 < *P* ≤ 0.05, **0.001 < *P* ≤ 0.01, ***P ≤ 0.001

A correlation analysis of the bacteria in the enrichment culture and the P200 metabolites was conducted using the Spearman correlation method (Fig. 3). The results showed that many metabolites were significantly correlated with community composition (family level). In detail, dihydrothymine was positively correlated with the families *Anaerolineaceae* and *Clostridiaceae*_3. 5-amino-4-carbamoylimidazole (AICA) and 4-pyridoxic acid were positively correlated with the families *Clostridiaceae*_1 and MSBL8, respectively. The family *Thermoanaerobaculaceae*, however, showed positive correlations with the majority of metabolites.

### SCFAs

SCFAs, which are key fermentation products in organic matter degradation, are generated in primary biological fermentation and metabolised by sulphate-reducing bacteria, methanogens and other bacterial groups as substrates in secondary fermentation (Muyzer and Stams 2008). Studies have found that the concentrations of SCFAs in pore waters are controlled by the final mineralization process, which uses acetic acid as a substrate in processes such as iron reduction, sulphate reduction and methanogenesis reduction (Glombitza et al. 2019). The SCFA concentrations that are produced by microorganisms in turn affect the compositions of microbial communities (Glombitza et al. 2019). For example, *Flavobacteriales* and *Marinilabiliales* are mostly decomposers of macromolecular compounds (Morishige et al. 2013), and bacteria of the phylum *Kiritimatiellaeota* are almost always fermenting types (Sackett et al. 2019). Their abundances and metabolic activities affect the degradation efficiency of organic matter and increase the rate of SCFAs. Sulphate-reducing bacteria such as *Desulfobacterales* are generally the main contributor to the anaerobic mineralization of SCFAs. The abundances and the metabolic activities of these groups influence the depletion rate of SCFAs. These interactions promote the mineralization of organic matter in marine sediments.

**Fig. 4:**
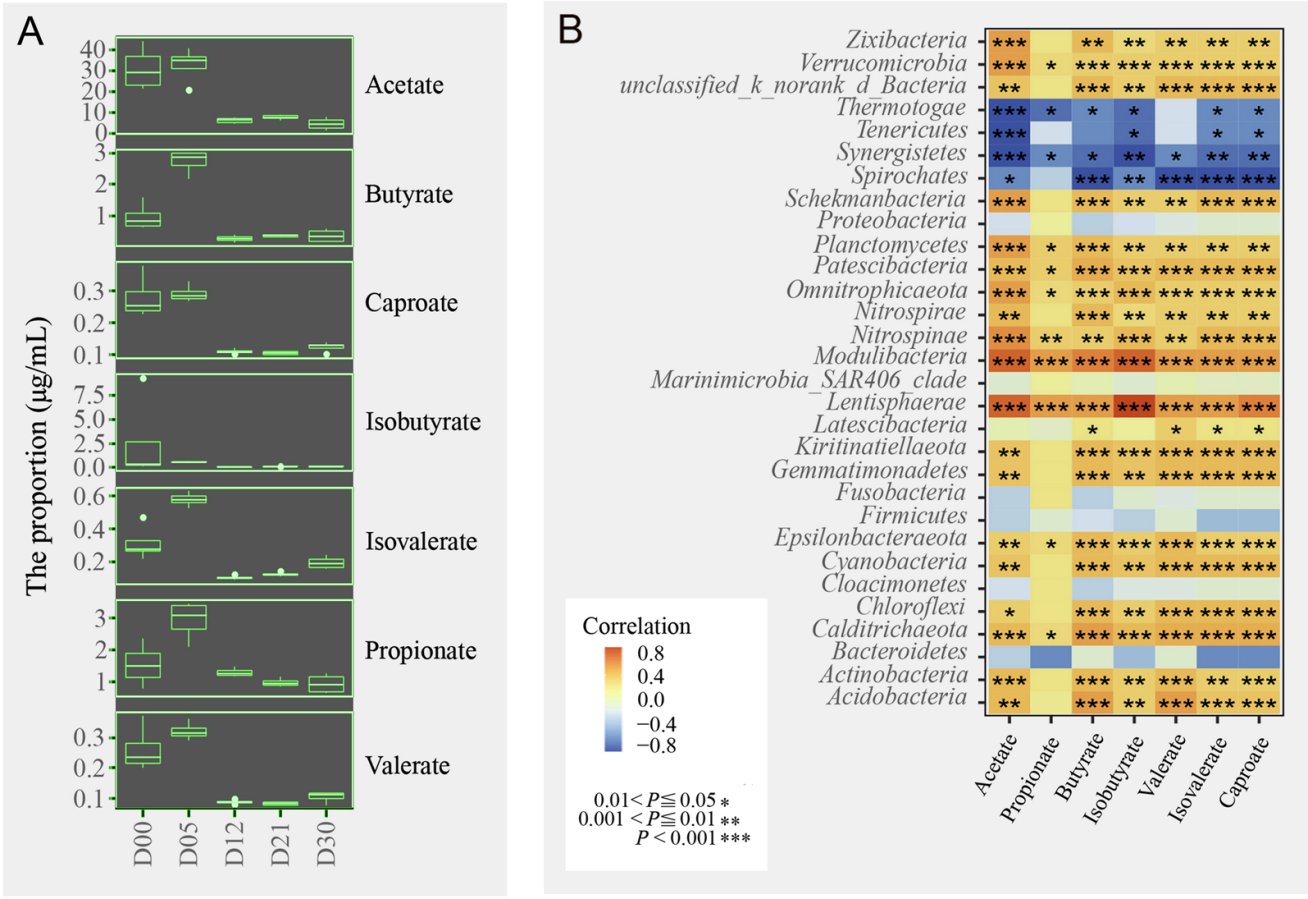
Correlations amongst community compositions and SCFAs. **A** Ranges of SCFA concentrations in different incubation stages. **B** Pearson correlations amongst different phyla in all incubation stages and SCFAs. The gradient colours represent the different correlations. The black points are within the abundant taxa community, and the red points are within the rare taxa community in all incubation stages. *0.01 < *P* ≤ 0.05, **0.001 < *P* ≤ 0.01, ***P ≤ 0.001

GC‒MS/LC‒MS was used to measure the SCFAs in microbial extracellular metabolites during different enrichment culture periods. ANOVA was used to analyse the SCFA data to detect significant differences in SCFAs between samples (Fig. 4A). The results showed that SCFA content increased significantly during the progress of the enrichment culture. The SCFA metabolites increased significantly, especially in D05, whilst the isobutyric acid, hexanoic acid, valeric acid, butyric acid and propanoic acid contents nearly all increased. This is presumably because during the enrichment culture, the bacterial groups producing butyric acid and propionic acid all increased. With the progression of the enrichment culture, different kinds of SCFAs showed different rates of increase and decrease, no doubt because of their complex reactions and associations. Acetic acid levels decreased on D05 and D12, increased on D21 and then decreased again on D30. Propanoic acid and butyric acid levels increased on D05 and D12, decreased on D21, and then increased again on D30. The isobutyric acid, valeric acid, isovaleric acid and hexanoic acid contents showed similar variation trends with relatively low concentrations. Acetate propionate and butyrate were the main SCFA components. As the enrichment culture proceeded, the added nutrients were constantly consumed by the bacteria. The nutrients were composed not only of carbohydrates and lipids but also of protein. Peptone is an important organic substance in enrichment media. In anaerobic environments, proteins are degraded much more slowly than carbohydrates (Yang et al. 2015). Under anaerobic conditions, some proteins are hydrolysed to peptides and amino acids, are subsequently fermented to short-chain fatty acids, and are finally converted to methane and carbon dioxide by specific microorganisms. Therefore, in the early enrichment stages, such as D05, with the degradation of macromolecules, the SCFA levels increased significantly. At the same stage, the number of acid-producing bacteria also increased significantly. As described above, the significant increases in the relative abundances of bacterial groups in *Firmicutes* and *Fusobacteria*, and especially the groups in *Clostridiales* (Rombouts et al. 2020), produced an increase in SCFAs. Studies have shown that *Clostridial clusters* IV and XIVa, which are highly sensitive to oxygen, produce the highest number of butyric acid bacteria in human faeces (Maia et al. 2007). In addition, *Lactobacillus* can produce propionic acid (Mamuad et al. 2017), and *Smithella propionica* can produce acetate and butyrate through disproportionation of propionate, which is finally degraded to acetate (de Bok et al. 2001).

In the late enrichment stages, the microbial community structure was further changed, for example, in the increased abundances of bacteria relying on SCFAs, which resulted in reductions in SCFA concentrations. Degradation of SCFAs is usually catalysed by SCFA-oxidising bacteria such as sulphate-reducing bacteria in marine sediments. SCFAs serve not only as carbon sources but also as electron donors for sulphate-reducing bacteria, which play an important role in the conversion of SCFAs in anaerobic condition (e.g. butyric acid and propionic acid) to acetate, H_2_ and formate. Studies have shown that isovalerate or isobutyrate is required for growth of the coccus, *Ruminococcus flavefaciens *(Allison et al. 1962). Isobutyric acid, and/or isovaleric acid and/or 2-methylbutyric acid can stimulate the growth of cellulolytic cocci isolated from rumen fluid (Kim and Bae 2016). These results suggest that SCFAs can be used as growth factors to stimulate microbial growth.

Correlations between the SCFAs from metabolites and bacteria at different enrichment stages underwent a Spearman analysis (Fig. 4B). The results showed that at the phylum level, there were no significant correlations of SCFAs metabolites with the predominant bacterial taxa (e.g. *Bacteroidetes*, *Proteobacteria*, *Firmicutes* and *Fusobacteria*), but correlations between SCFAs and multiple uncultured bacterial groups were significant. For example, *Chloroflexi*, *Planctomycetes*, *Kiritimatiellaeota* and *Verrucomicrobia* showed significant positive correlations, whilst *Spirochaetes* and *Synergistetes* showed significant negative correlations.

### SCFAs facilitate the isolation of uncultured strains

These above results might imply that it might be possible to obtain rare taxa when supplying some SCFAs and metabolites. Thus, clarification is required as to whether the SCFAs and metabolites in the culture medium facilitate the isolation of uncultured bacterial groups by providing the same nutrition conditions. The extracellular metabolites produced during the enrichment culturing were so complicated that the RCA medium was selected to simulate them. The RCA medium contained amino acids and horse serum (with a variety of growth factors). Amino acids and growth factors were also detected in the previous test for metabolites. The SCFAs were simulated by adding SCFAs to the culture media. The SCFAs were of interest because they showed significant correlations with rare taxa, and it was thought they might be important intermediate metabolites and be directly related to energy use as carbon sources.

RCA medium with (experimental group) or without (CK group) SCFAs was used as the isolation culture medium to isolate and culture the uncultured bacteria. Isolation was carried out after each enrichment period, and the bacteria were cultured under anaerobic conditions for 30 days with or without addition of SCFAs. A total of 444 strains of bacteria were isolated under anaerobic conditions after five enrichment culture periods, of which 206 strains (14 of them were candidate novel species without duplicates) were isolated from the CK group and 238 strains were isolated from the experimental group (50 of them were candidate novel species without duplicates). The isolated strains belonged to 10 phyla, 28 orders, 42 families and 133 species.

Through 16S rRNA gene sequencing analysis, it was determined that the abundance and the diversity of candidate novel species (16S rRNA gene similarity < 98.65%) (Kim et al. 2014) that were isolated in the experimental group (with SCFAs) were lower than those in the CK group (without SCFAs) in the D00 period (Table 1). In the other enrichment periods, the number of candidate novel species that were isolated in the experimental group was much higher. In addition, the candidate novel species that were isolated from the CK group in the five enrichment culture periods totaled 57 strains (sum of numbers in brackets for the CK group), whilst 44 of them were duplicates.

**Table 1:** Number of cultured bacteria and candidate novel species

	D00	D05	D12	D21	D30
CK group	23 (9)	46 (4)	58 (21)	38 (13)	41 (10)
Experimental group	18 (4)	45 (9)	63 (28)	48 (21)	64 (19)

The numbers before the parentheses represent cultured bacteria in different enrichment stages, and the numbers in parentheses represent the candidate novel species

**Fig. 5:**
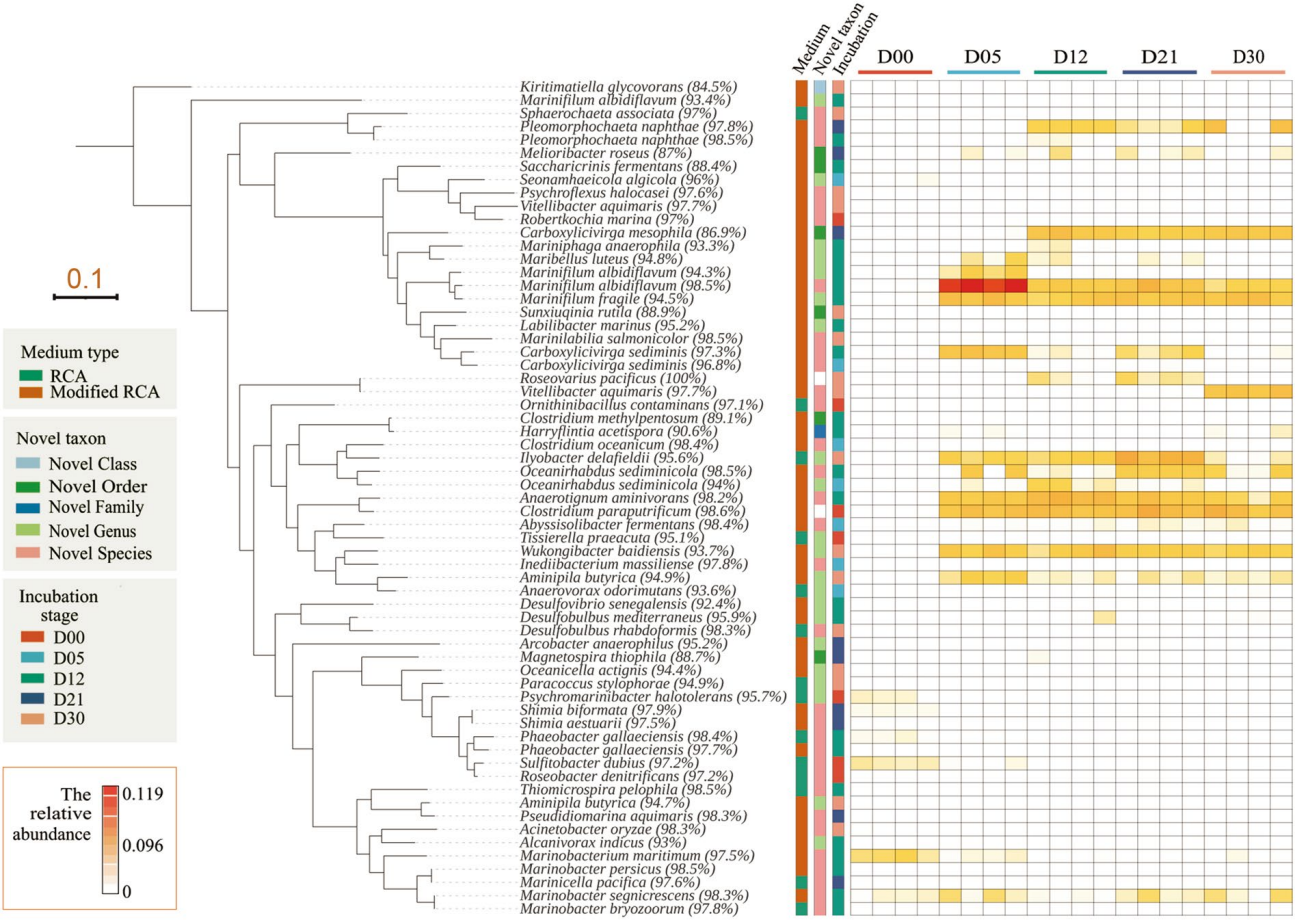
Phylogenetic analysis of isolated candidate novel species. The ML phylogenetic tree is displayed in a rectangular layout with a tree scale of 0.1. The first strip on the right of the tree represents the two different media types of isolated strains. The second strip represents the candidate taxon levels. The third strip represents the incubation stages of the isolated strains, and the different incubation stages are randomly coloured. The heatmap represents the relative abundances of isolated strains in the high-throughput sequencing results of the 16S rRNA gene dataset

After the five periods in the enrichment culture, 49 genera and 69 species were isolated from the CK group, and 68 genera and 82 species were isolated from the experimental group (Table 2). The bacteria that were isolated from the two groups of media were significantly different. Only 20% of the total of 97 genera were isolated from both groups indicating the large difference in bacterial groups that were isolated from the two media at the genus level. The initial abundances of all isolated strains were less than 0.1%. Fourteen of 64 strains were enriched to over 0.1%, and eight strains were enriched to approximately 1%. Only *Marinifilum albidiflavum* (98.5%) was enriched to over 5% (11.9%) (D05) (Fig. 5). In addition, several uncultured bacteria were successfully isolated on the experimental group medium. For example, *Chlorobi* and *Kiritimatiellaeota* showed significant correlations with SCFAs between metabolites and bacteria groups. The experimental group not only increased the diversity of culturable bacteria and number of new bacteria but also benefited the culture of low-abundance bacteria. Therefore, it was presumed that the addition of SCFAs to the culture medium could change the nutrient conditions of traditional media and then had a significant effect on the diversity of culturable bacteria.

**Table 2:** Analysis of cultured bacteria in different media

	Bacteria number	phylum	Family	Species	Candidate novel species	16S rRNA gene similarity < 90%
CK group	206	6	31	69	14	0
Experimental group	238	10	26	82	50	8

**Fig. 6:**
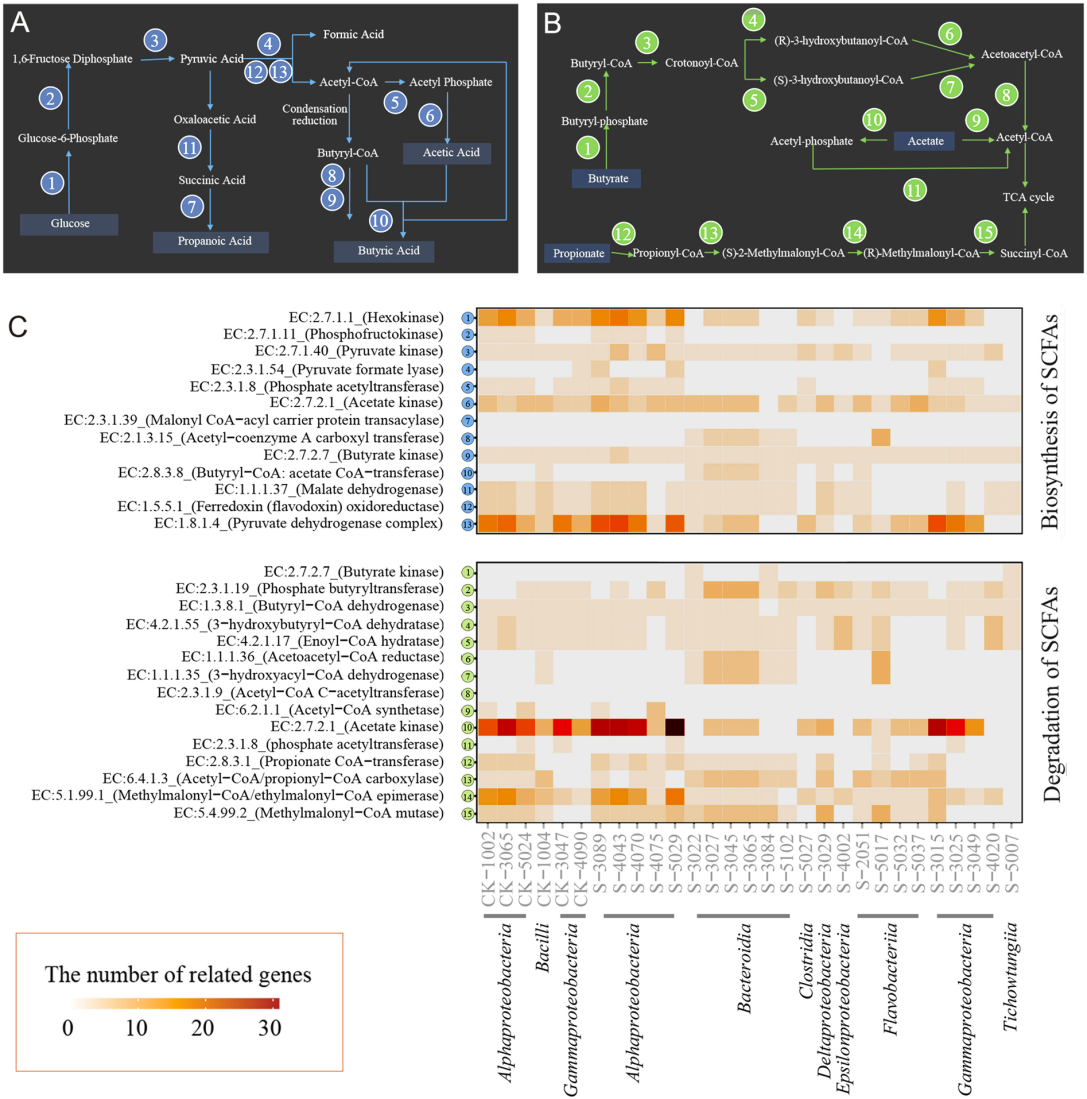
Reconstruction of SCFA synthesis and degradation in candidate novel species. **A**–**B** Schematic diagram of SCFA synthesis and degradation pathways. The numbers in circles represent the enzymes in the pathways. **C** Reconstruction of SCFA synthesis and degradation pathways based on 29 isolated strains. The heatmap represents the number of related genes in the SCFA synthesis and degradation pathways

The adoption of microbial functions is highly correlated with the expression of functional genes (Fang et al. 2022). To determine the reason for the isolation of uncultured groups with the addition of SCFAs, the genomes of 29 candidate novel species were detected and analysed. The genomic sequencing results of the candidate novel species for the SCFA biosynthetic and metabolic pathways are shown in Fig. 6. In two pathways, each reaction step is completed by individual enzyme catalysis (Magnuson et al. 1993), and each enzyme is encoded by its specific gene.

In anoxic marine sediments, the microbial degradation of organic macromolecules is a complex process (Pelikan et al. 2021). The “primary degraders” break down larger macromolecules into oligomers and monomers for subsequent fermentation to alcohols, lactate and/or SCFAs, which are then mineralized to CH_4_, CO_2_ and/or H_2_ (Muyzer and Stams 2008). The simplified synthetic SCFA pathway is shown in Fig. 6A. Glucose is the most commonly used carbon source. Under anaerobic conditions, glucose is first converted to pyruvic acid through the glycolytic pathway and then to acetyl-CoA by pyruvate formate-lyase with the release of CO_2_ (Zhang et al. 2019). In the acid-forming process, phosphate acetyltransferase and acetate kinase (Yuan et al. 2016) are responsible for the formation of acetic acid. Propionic acid is converted from pyruvic acid by malate dehydrogenase and malonyl-CoA-acyl carrier protein transacylase. Acetyl-CoA butyryl-CoA is first converted to butyryl-CoA and then converted to butyric acid through butyrate kinase and phosphotransbutyrylase without acetic acid (Miller and Jenesel 1979) and through butyryl-CoA:acetate CoA-transferase with acetic acid (Diez-Gonzalez et al. 1999; Duncan Sylvia et al. 2002).

In the genomic data for the synthesis of SCFAs (Fig. 6C), it was noted that almost all the candidate novel species in the CK group could synthesise acetic acid and butyric acid, whilst all the candidate novel species lacked the key gene for the synthesis of propionic acid, namely, malonyl-CoA  acyl carrier protein transacylase. However, most of the candidate novel species in the experimental group lacked phosphofructokinase, a key gene in the glucose-based SCFA synthesis pathway that enables SCFA biosynthesis. Therefore, it is speculated that SCFA metabolism in the enrichment culture was mainly based on SCFA utilisation and the addition of SCFA in the isolation culture may have affected the emergence of some groups.

The utilisation of SCFAs as carbon sources can be implemented through coenzyme A attachment and subsequent oxidation (Trautmann et al. 2020). As shown in Fig. 6B, there are two pathways for the conversion of acetate to acetyl-CoA: (1) the reversible acetate kinase-phosphate acetyltransferase pathway and (2) the irreversible pathway catalysed by acetyl-CoA synthetase (Zhang et al. 2019). Butyric acid is catalysed into butyryl-CoA and then into acetyl-CoA via butyrate kinase and phosphate butyryltransferase. Propionyl-CoA is catalysed by propionate CoA  transferase and then converted to succinyl-CoA. Acetyl-CoA and succinyl-CoA then enter the TCA cycle to produce energy.

Acetyl-CoA is an important metabolic intermediate, which is the substrate of fatty acid synthesis and the product of fatty acid degradation. In addition to its important roles in the TCA cycle, acetyl-CoA also serves as an acetylation precursor for the biosynthesis of various acetyl chemicals, such as ketones, cholesterol and steroid compounds. Although glucose is an efficient carbon source for acetyl-CoA production, the pathway from acetate to acetyl-CoA is the shortest and SCFAs can produce acetyl-CoA through fatty acid oxidation along with abundant NADH and FADH_2_ (Zhang et al. 2019). Instead of acetyl-CoA, SCFA-CoA can be transferred to malonyl-CoA (Fujita et al. 2007). Therefore, SCFA-CoA forms the primer molecule to convert long-chain fatty acids (Trautmann et al. 2020).

In addition to being utilised as carbon sources, SCFAs are also available electron donors in marine sediments. When SCFAs are available, sulphate-reducing microorganisms (SRM) known as terminal oxidizers of fermentation products (Finke et al. 2007; Glombitza et al. 2015, 2019), can achieve the final mineralization of organic matter via oxidation of SCFAs.

In the genomic data for SCFA utilisation, it was found that some of the candidate novel species contained genes (e.g. acetyl  CoA synthetase or acetate kinase and phosphate acetyltransferase) that are involved in breaking down acetic acid and almost all potential new bacteria can decompose propionic acid to succinyl-CoA through a series of enzymes (e.g. propionate CoA  transferase, acetyl  CoA/propionyl  CoA carboxylase, methylmalonyl  CoA/ethylmalonyl  CoA epimerase and methylmalonyl  CoA mutase), which enter into TCA cycles to produce energy. Most candidate novel species cannot utilise butyric acid due to a lack of butyrate kinase, a key gene for the decomposition of butyric acid.

After analysing the above results, it was speculated that most of the bacteria that were isolated from the CK group could biosynthesize SCFAs, which indicates that the hydrolysis and fermentation of macromolecular organic matter may be a common process in the original samples. As the enrichment proceeded, SCFAs were continuously produced, and microorganisms that can utilise or metabolise SCFAs were activated. This activation process may be accompanied by a transition from dormancy to recovery.

It is speculated that the addition of SCFAs played an important role in the isolation culture of uncultured bacteria groups after the enrichment culture process. SCFAs may serve as available carbon sources that are more readily used by some microorganisms to promote microbial growth in the enrichment culture. As discussed above, the abundance of most of the isolated strains was lower than 0.1% during the enrichment culture (Fig. 5), which indicated that the SCFAs did not increase the abundances of the isolated strains but altered the state of the strains. In addition, it was found that the isolated candidate novel species (e.g. *Kiritimatiellaeota*, *Planctomycetes* and *Marinilabiliales*) in the SCFA growth-dependent experiment could also grow on the culture without SCFAs and show no significant difference (naked eye) in growth state after they were successfully isolated, indicating that the SCFAs were not their growth factor but that the addition of SCFAs could promote the formation of their growth curves.

Twelve *Marinilabiliales* strains were isolated from the experimental group and eight of them were. The 16S rRNA gene similarities of three strains [e.g. *Saccharicrinis fermentans* (88.4%), *Carboxylicivirga mesophila* (86.9%) and *Sunxiuqinia rutila* (88.9%)] were lower than 90%. The representative characteristics of this group are facultative aerobic or anaerobic, capable of fermentation and metabolism (Wu et al. 2016) and a strong ability to degrade macromolecular compounds (Shalley et al. 2013). It is speculated that the addition of SCFAs to the culture medium may be important to increasing the diversity of *Marinilabiliales*. As described above, the isolated candidate novel species were growth-independent of SCFAs, which could be assumed to prompt them from a dormant state into the active state or to shorten the stagnation period of bacterial growth, thus enabling them to adapt to the medium environment more quickly.

**Fig. 7:**
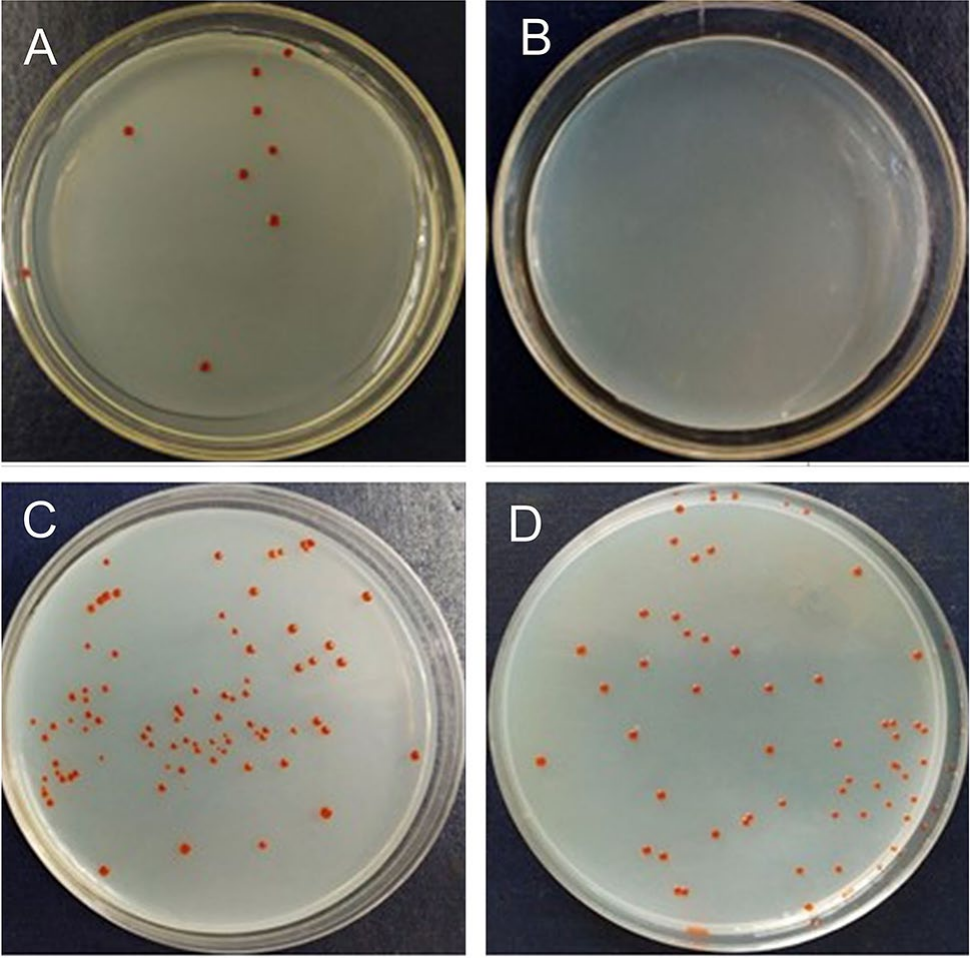
Resuscitation culture. **A** Colony morphology plot of *Marinilabilias salmonicolor* underlined cultures after 5 days (**A**) with SCFAs (**B**) without SCFAs and 12 days enrichment (**C**) with SCFAs (**D**) without SCFAs

To verify this assumption, a strain of *Marinilabilias salmonicolor* was introduced into the VBNC state and then recovered with SCFAs. A previous meta-transcriptomics study showed that *Marinilabilias* bacteria demonstrated a shift in metabolic level from low to high (Mu et al. 2018), which was considered the breaking of dormancy. Because *Marinilabilias salmonicolor* was isolated repeatedly in the experimental group was easily cultured, it was chosen to carry out the dormancy and recovery test to verify the assumption regarding the recovery effect of SCFAs. *Marinilabilias salmonicolor* was induced into dormancy by cryogenic oligotrophic stress. The strain was stored at 4 ℃ for 98 days (Supplementary Fig. S1) and then entered into VBNC state. At this time, no colonies were found on the plate after coating. The viability of the bacteria was confirmed using LIVE/DEAD BacLight Bacterial Viability Kit (ThermoFisher L7012). SYTO 9 stained all cells green, and no cells stained red by propidium iodide were seen in the same field of view. This indicates that dormant cells remained alive (Supplementary Fig. S2). The enrichment cultures with/without SCFAs were designed to culture dormant *Marinilabilias salmonicolor* under anaerobic conditions*.* Spread plate cultures were performed and Fig. 7A shows that there some colonies of the *Marinilabilias salmonicolor* strain were present in the medium with SCFAs on Day 5, and that there were no significant colonies in the medium without SCFAs, showing higher efficient recovery of SCFAs. On Day 12, there were more colonies in the medium with SCFAs than in the medium without SCFAs. Thus, it can be concluded that SCFAs have a recovery effect on dormant bacteria.

**Fig. 8:**
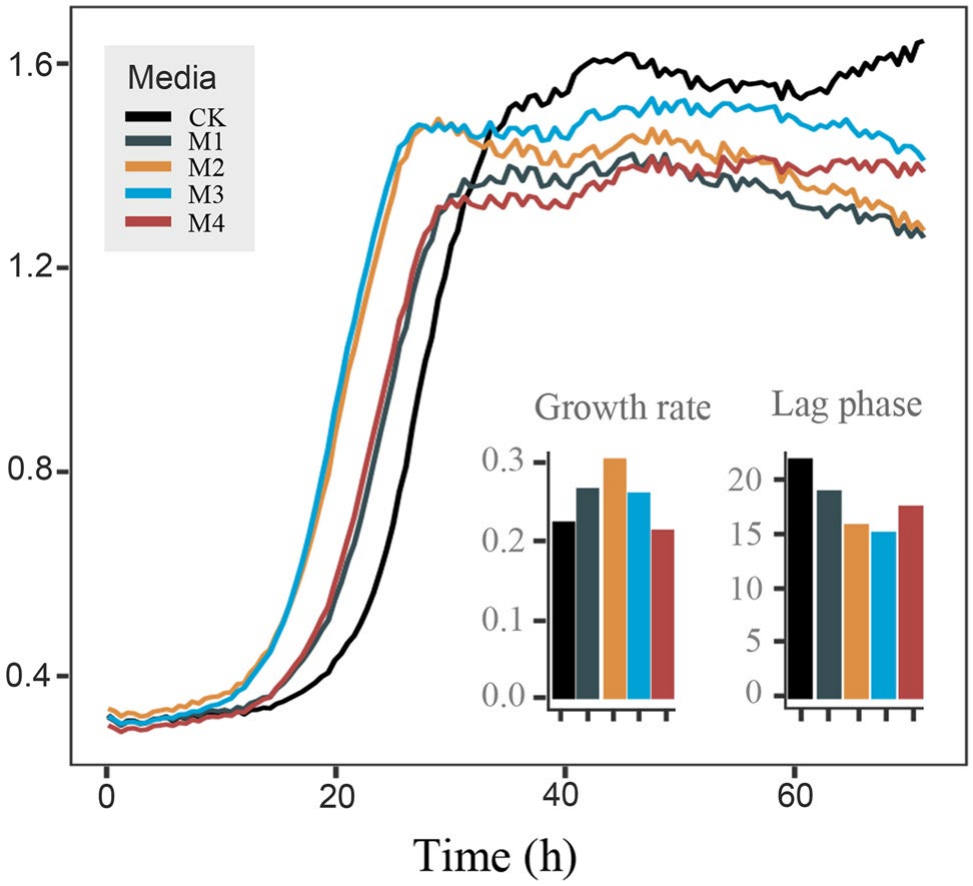
Growth curve test. Growth of *Marinilabilias salmonicolor* under different culture conditions. The bacterial growth curves represent *Marinilabilias salmonicolor* strains under different culture conditions. CK: 1/10 2216E, M1: 1/10 2216E + CH_3_COONa, M2: 1/10 2216E + C_3_H_5_O_2_Na, M3: 1/10 2216E + C_4_H_7_O_2_Na, and M4: 1/10 2216E CH_3_COONa + C_3_H_5_O_2_Na + C_4_H_7_O_2_Na

To further determine the recovery mechanism, a growth curve of *Marinilabilias salmonicolor* was produced by adding SCFAs to the culture medium. *Marinilabilias salmonicolor* was cultured at 4 ℃ for 7 days to stop the growth by low-temperature stress. The growth curves were measured by a growth curve metre with different media containing different SCFAs. It can be seen in Fig. 7B that compared with the CK group, all the media with SCFAs shortened the lag phase. Through fitting calculations, it was determined that propionic acid and butyric acid shortened the lag phase to approximately 15 h, and the lag phase of the CK group to more than 20 h. SCFAs also increased the growth rate of the bacteria, and propionic acid had the best effect. As had previously been determined, all the candidate novel species that were isolated had the gene to degrade propionic acid (Fig. 8).

## Conclusion

In conclusion, our results provide the first insight into the effect of SCFAs as resuscitation factors on facilitating the culture efficiency of uncultured bacteria in marine sediments and demonstrated that the SCFAs added into the medium would shorten the time of resuscitation culture of bacteria. As a result, SCFAs can be used as resuscitation factors to culture previously uncultured bacteria that were in the VBNC state in the marine environment. During the enrichment culture, it was deduced that the SCFAs determined from metabonomics promoted resuscitation of the VBNC bacteria because they showed significant correlations with multiple uncultured bacteria groups. During the isolated culture, it was deduced that adding SCFAs to the agar medium provided not only an energy source but also a resuscitation factor for bacteria. As a result, isolation of several strains of uncultured bacteria was successful. In further studies, an attempt will be made to intensively study the mechanisms for resuscitation culture of SCFAs.

## Materials and methods

### Marine sediment sampling and incubation

All coastal sediment samples were collected from Xiaoshi Island at Weihai, China (37° 31′ 36″ N, 122° 00′ 58″ E) in September 2018. Samples from depths of 0 to 15 cm were collected, placed in sterile plastic bags, kept cold within an ice box and immediately processed for incubation. The incubation medium is referenced from a previous study (Mu et al. 2018) and consisted of the following substances placed in natural seawater: 0.1% NH_4_Cl, 0.2% CH_3_COONa, 0.02% MgSO_4_·7H_2_O, 0.02% yeast extract, 0.02% peptone, 0.1% EDTA and 0.125% sodium pyruvate. The pH of the medium was adjusted to 7.0, and it was then autoclaved. Twenty millilitres of 5% (w/v) NaHCO_3_ solution and 10 mL of 2% (w/v) KH_2_PO_4_ solution were filtered and added to the autoclaved media. Twenty grammes of sediment was added to 250 mL sealed glass bottles, which were then filled with approximate amounts of incubation medium. All bottles were stored at 33 °C for 0, 5, 12, 21 and 30 days and shaken twice daily. In total, 20 samples were collected and stored at − 80 °C for later analysis.

### Measurement of physical–chemical factors and metabolomics

Four replicates were selected at each incubation time point to measure physical–chemical factors, short-chain fatty acids (SCFAs) and targeted metabolomics. The supernatants were collected after centrifugation (12,000 r/min × 15 min, 4 °C) and stored at − 80 °C. The physical–chemical factors were measured at the Scientific Instruments Sharing Platform, Third Institute of Oceanography, Ministry of Natural Resources (http://www.tio-sisp.net/JMISP/, Xiamen China) using standard testing methods. SCFAs and targeted metabolomics were detected at Shanghai Applied Protein Technology Co., Ltd. (http://www.aptbiotech.com/, China).

### DNA extraction, 16S rRNA gene sequencing and analysis

The microbial community genomic DNA from samples was obtained using the E.Z.N.A.^®^ soil DNA Kit (Omega Bio-tek, Norcross, GA, U.S.) according to the manufacturer’s protocols. The 16S rRNA genes (e.g. V3–V4) were amplified using general primers (e.g. 338F/806R). Sequencing was performed on the Illumina MiSeq PE300 platform (Illumina, San Diego, USA) according to the standard protocols by Shanghai Majorbio Biopharm Technology Co. Ltd. (Shanghai, China). Paired-end reads were merged by FLASH version 1.2.11 (Magoč and Salzberg 2011). A total of 700,820 high-quality reads were obtained from 20 samples, and the lowest number of reads amongst the samples, 35,041, was chosen to rarefy the datasets for all community comparisons. Operational taxonomic units (OTUs) with a 97% similarity cutoff were clustered using UPARSE version 7.0 (Edgar 2013), and chimeric sequences were identified and removed. The taxonomy of each representative OTU sequence was analysed by RDP Classifier version 2.11 (Wang et al. 2007) against the Silva v138 16S rRNA database (Quast et al. 2013).

The rank abundance distributions (RADs) for each sample were calculated using the R package “RAD analysis” (Saeedghalati et al. 2017). NMDS analysis was performed using the R package “vegan” (Dixon 2003) based on Bray–Curtis dissimilarities. Nonparametric multivariate statistical analyses [Adonis, analysis of similarity (ANOSIM) and multi-response permutation procedure (MRPP)] based on Bray–Curtis distances were used to assess whether the bacterial community compositions were different. The differential abundance tests of the microbial communities across groups were analysed based on LEfSe (Segata et al. 2011). To explore the community structural–functional relationships, Tax4Fun2 (Wemheuer et al. 2020) was used to predict the possible functional profiles and functional redundancy.

### Isolation and classification of bacteria during incubation

Serial dilutions of the incubation samples were spread-plated onto two rich media (e.g. RCA media and modified RCA media). The plates were then incubated at 30 °C for 30 days in anaerobic jars (10% H_2_, 10% CO_2_ and 80% N_2_). The following growth media were used in this study: RCA supplemented with 2.4 g/L beef extract, 3.0 g/L yeast extract, 4.0 g/L Na_2_HPO_4_, 1.5 g/L glucose, 0.5 g/L starch, 0.5 g/L L-cysteine monohydrochloride, 50 mL of normal horse serum and 15 g/L agar with artificial sea water. The modified RCA was supplied with five SCFAs (e.g. CH_3_COONa, C_3_H_5_O_2_Na, C_4_H_7_O_2_Na, C_5_H_10_O_2_ and C_6_H_12_O_2_) to a concentration of 1.0 mmol/L on the basis of RCA.

The 16S rRNA gene was amplified by PCR with the forward primer 27F and the reverse primer 1492R (Liu et al. 2014). Purified PCR products were sequenced by BGI Co. Ltd (Qingdao, PR China) and resulted in about 1350 bp nearly complete 16S rRNA gene sequence. Pairwise similarity values were calculated using a blast sequence similarity search (Altschul et al. 1990) and the EzBioCloud server (www.ezbiocloud.net) (Yoon et al. 2017) to identify the nearest taxa.

### Genomic reconstruction of SCFA synthesis and degradation

To explore the potential capacity of SCFA synthesis and degradation, 29 draft genomes of the isolated bacterial strains were sequenced. The genomic DNA of all strains was extracted with a DNA extraction kit (TaKaRa Bio) according to the manufacturer’s instructions. Draft genome sequencing was performed by Shanghai Personal Biotechnology Co., Ltd. (Shanghai, China) using an Illumina PE150 system. The final genomes were assembled using SOAPdenovo version 2.04 (https://www.genomics.cn/). The genome-based metabolic potential capacities of the isolated strains were predicted using the RAST server (https://rast.nmpdr.org/).

### Induction of VBNC state and live/dead staining

*Marinilabilias salmonicolor* was cultured on marine agar 2216 (MA; BD) to the logarithmic phase for 3 h with mild shaking at 150 r/min, and harvested by centrifugation at 16,000×*g* for 3 min. The cells were washed 5 times with sterile artificial seawater and finally re-suspended in sterile artificial seawater to a density of 108 CFU/mL. The cells were then stored at 4 °C for 98 days. The bacterial cell state was monitored over time using the dilution plate count method on MA medium. VBNC cells were defined as bacteria that could not form colonies on MA after such treatment. VBNC cells were incubated in enrichment culture medium at 25 °C, spread onto MA medium and incubated at 25 °C for 1 day. The viability of the bacteria was confirmed using LIVE/DEAD BacLight Bacterial Viability Kit (ThermoFisher L7012).

## Supplementary Information

Below is the link to the electronic supplementary material.

Supplementary file1 (DOCX 3088 KB)

## Data Availability

The data that support the findings of this study are included in this published article (and its supplementary information file).

## References

[CR1] Allison MJ, Bryant MP, Katz I, Keeney M (1962). Metabolic function of branched-chain volatile fatty acids, growth factors for ruminococci ii: biosynthesis of higher branched-chain fatty acids and aldehydes. J Bacteriol.

[CR2] Altschul SF, Gish W, Miller W, Myers EW, Lipman DJ (1990). Basic local alignment search tool. J Mol Biol.

[CR3] Colwell RR (2000). Viable but nonculturable bacteria: a survival strategy. J Infect Chemother.

[CR4] de Bok FA, Stams AJ, Dijkema C, Boone DR (2001). Pathway of propionate oxidation by a syntrophic culture of *Smithella propionica* and *Methanospirillum hungatei*. Appl Environ Microbiol.

[CR5] Debnath A, Miyoshi SI (2021). The impact of protease during recovery from viable but non-culturable (vbnc) state in vibrio cholerae. Microorganisms.

[CR6] Diez-Gonzalez F, Bond DR, Jennings E, Russell JB (1999). Alternative schemes of butyrate production in butyrivibrio fibrisolvens and their relationship to acetate utilization, lactate production, and phylogeny. Arch MIicrobiol.

[CR7] Dixon P (2003). Vegan, a package of r functions for community ecology. J Veg Sci.

[CR8] Duncan Sylvia H, Barcenilla A, Stewart Colin S, Pryde Susan E, Flint Harry J (2002). Acetate utilization and butyryl coenzyme a (coa): acetate-coa transferase in butyrate-producing bacteria from the human large intestine. Appl Environ Microbiol.

[CR9] Edgar RC (2013). Uparse: highly accurate otu sequences from microbial amplicon reads. Nat Methods.

[CR10] Fang SY, Guo W, Feng Q, Cao WB, Dou YT, Huang WX, Wang F, Cheng XS, Cao J, Wu Y, Luo JY (2022). Surfactants aggravated the biotoxicity of fe2o3 nanoparticles in the volatile fatty acids’ biosynthesis during sludge anaerobic fermentation. ACS ES&T Water.

[CR11] Finke N, Vandieken V, Jørgensen BB (2007). Acetate, lactate, propionate, and isobutyrate as electron donors for iron and sulfate reduction in arctic marine sediments, Svalbard. FEMS Microbiol Ecol.

[CR12] Fujita Y, Matsuoka H, Hirooka K (2007). Regulation of fatty acid metabolism in bacteria. Mol Microbio.

[CR13] Glombitza C, Jaussi M, Røy H, Seidenkrantz MS, Lomstein BA, Jørgensen BB (2015). Formate, acetate, and propionate as substrates for sulfate reduction in sub-arctic sediments of southwest greenland. Front Microbiol.

[CR14] Glombitza C, Egger M, Røy H, Jørgensen BB (2019). Controls on volatile fatty acid concentrations in marine sediments (Baltic sea). Geochim Cosmochim Acta.

[CR15] Jung DW, Machida K, Nakao Y, Owen JS, He S, Kindaichi T, Ohashi A, Aoi Y (2022). Cultivation of previously uncultured sponge-associated bacteria using advanced cultivation techniques: a perspective on possible key mechanisms. Front Mar Sci.

[CR16] Kaprelyants A, Kell DB (1993). Dormancy in stationary-phase cultures of micrococcus luteus: flow cytometric analysis of starvation and resuscitation. Appl Environ Microbiol.

[CR17] Kim MS, Bae JW (2016). Spatial disturbances in altered mucosal and luminal gut viromes of diet-induced obese mice. Environ Microbiol.

[CR18] Kim M, Oh H-S, Park S-C, Chun J (2014). Towards a taxonomic coherence between average nucleotide identity and 16s rRNA gene sequence similarity for species demarcation of prokaryotes. Int J Syst Evol Microbiol.

[CR19] Lennon JT, Jones SE (2011). Microbial seed banks: The ecological and evolutionary implications of dormancy. Nat Rev Microbiol.

[CR20] Li SH, Jin Y, Cheng J, Park DJ, Kim CJ, Hozzein WN, Wadaan MAM, Shu WS, Ding LX, Li WJ (2014). *Gordonia jinhuaensis* sp. Nov., a novel actinobacterium, isolated from a vbnc (viable but non-culturable) state in pharmaceutical wastewater. Anton Leeuw Int J G.

[CR21] Liang QY, Zhang JY, Ning DL, Yu WX, Chen GJ, Tao XY, Zhou JZ, Du ZJ, Mu DS (2023). Niche modification by sulfate-reducing bacteria drives microbial community assembly in anoxic marine sediments. Mbio.

[CR22] Liu Q-Q, Li X-L, Rooney AP, Du Z-J, Chen G-J (2014). *Tangfeifania diversioriginum* gen. Nov., sp. Nov., a representative of the family draconibacteriaceae. Int J Syst Evol Microbol.

[CR23] Magnuson K, Jackowski S, Rock CO, Cronan JE (1993). Regulation of fatty acid biosynthesis in *Escherichia coli*. Microbiol Res.

[CR24] Magoč T, Salzberg SL (2011). Flash: fast length adjustment of short reads to improve genome assemblies. Bioinformatics (oxford, England).

[CR25] Maia MR, Chaudhary LC, Figueres L, Wallace RJ (2007). Metabolism of polyunsaturated fatty acids and their toxicity to the microflora of the rumen. Anton Leeuw Int J G.

[CR26] Mamuad LL, Kim SH, Choi YJ, Soriano AP, Cho KK, Lee K, Bae GS, Lee SS (2017). Increased propionate concentration in lactobacillus mucosae-fermented wet brewers grains and during in vitro rumen fermentation. J Appl Microbiol.

[CR27] Miller TL, Jenesel SE (1979). Enzymology of butyrate formation by butyrivibrio fibrisolvens. J Bacteriol.

[CR28] Morishige Y, Fujimori K, Amano F (2013). Differential resuscitative effect of pyruvate and its analogues on VBNC (viable but non-culturable) salmonella. Microbes Environ.

[CR29] Mu DS, Liang QY, Wang XM, Lu DC, Shi MJ, Chen GJ, Du ZJ (2018). Metatranscriptomic and comparative genomic insights into resuscitation mechanisms during enrichment culturing. Microbiome.

[CR30] Mu DS, Ouyang Y, Chen GJ, Du ZJ (2021). Strategies for culturing active/dormant marine microbes. Mar Life Sci Technol.

[CR31] Muyzer G, Stams AJM (2008). The ecology and biotechnology of sulphate-reducing bacteria. Nat Rev Microbiol.

[CR32] Nikitushkin VD, Demina GR, Shleeva MO, Kaprelyants AS (2013). Peptidoglycan fragments stimulate resuscitationof “non-culturable” mycobacteria. Anton Leeuw Int J G.

[CR33] Oliver JD (2010). Recent findings on the viable but nonculturable state in pathogenic bacteria. FEMS Microbiol Rev.

[CR34] Pelikan C, Wasmund K, Glombitza C, Hausmann B, Herbold CW, Flieder M, Loy A (2021). Anaerobic bacterial degradation of protein and lipid macromolecules in subarctic marine sediment. ISME J.

[CR35] Pinto D, Almeida V, Almeida Santos M, Chambel L (2011). Resuscitation of *Escherichia coli* VBNC cells depends on a variety of environmental or chemical stimuli. J Appl Microbiol.

[CR36] Quast C, Pruesse E, Yilmaz P, Gerken J, Schweer T, Yarza P, Peplies J, Glöckner FO (2013). The silva ribosomal RNA gene database project: improved data processing and web-based tools. Nucleic Acids Res.

[CR37] Rombouts JL, Kranendonk EMM, Regueira A, Weissbrodt DG, Kleerebezem R, van Loosdrecht MCM (2020). Selecting for lactic acid producing and utilising bacteria in anaerobic enrichment cultures. Biotechnol Bioeng.

[CR38] Sackett JD, Kruger BR, Becraft ED, Jarett JK, Stepanauskas R, Woyke T, Moser DP (2019). Four draft single-cell genome sequences of novel, nearly identical *kiritimatiellaeota* strains isolated from the continental deep subsurface. Microbiol Resour Ann.

[CR39] Saeedghalati M, Farahpour F, Budeus B, Lange A, Westendorf AM, Seifert M, Küppers R, Hoffmann D (2017). Quantitative comparison of abundance structures of generalized communities: from b-cell receptor repertoires to microbiomes. PLoS Comput Biol.

[CR40] Segata N, Izard J, Waldron L, Gevers D, Miropolsky L, Garrett WS, Huttenhower C (2011). Metagenomic biomarker discovery and explanation. Genome Biol.

[CR41] Senoh M, Ghosh-Banerjee J, Ramamurthy T, Colwell RR, Miyoshi S, Nair GB, Takeda Y (2012). Conversion of viable but nonculturable enteric bacteria to culturable by co-culture with eukaryotic cells. Microbiol Immunol.

[CR42] Shalley S, Kumar SP, Srinivas TNR, Suresh K, Kumar PA (2013). *Marinilabilia nitratireducens* sp. Nov., a lipolytic bacterium of the family *Marinilabiliaceae* isolated from marine solar saltern. Anton Leeuw Int J G.

[CR43] Su XM, Xie MQ, Han Z, Xiao YY, Wang R, Shen CF, Hashmi MZ, Sun FQ (2023). Resuscitation-promoting factor accelerates enrichment of highly active tetrachloroethene/polychlorinated biphenyl-dechlorinating cultures. Appl Environ Microbiol.

[CR44] Trautmann A, Schleicher L, Deusch S, Gatgens J, Steuber J, Seifert J (2020). Short-chain fatty acids modulate metabolic pathways and membrane lipids in *Prevotella bryantii* b_1_4. Proteomes.

[CR45] Wang Q, Garrity GM, Tiedje JM, Cole JR (2007). Naive Bayesian classifier for rapid assignment of rRNA sequences into the new bacterial taxonomy. Appl Environ Microbiol.

[CR46] Wang FP, Li M, Huang L, Zhang X-H (2021). Cultivation of uncultured marine microorganisms. Mar Life Sci Technol.

[CR47] Wemheuer F, Taylor JA, Daniel R, Johnston E, Meinicke P, Thomas T, Wemheuer B (2020). Tax4fun2: prediction of habitat-specific functional profiles and functional redundancy based on 16s rRNA gene sequences. Environ Microbiome.

[CR48] Wu WJ, Zhao JX, Chen GJ, Du ZJ (2016). Description of *Ancylomarina subtilis* gen. Nov., sp. Nov., isolated from coastal sediment, proposal of *Marinilabiliales* ord. Nov. And transfer of *Marinilabiliaceae*, *Prolixibacteraceae* and *Marinifilaceae* to the order *Marinilabiliales*. Int J Syst Evol Microbiol.

[CR49] Yang G, Zhang P, Zhang G, Wang Y, Yang A (2015). Degradation properties of protein and carbohydrate during sludge anaerobic digestion. Bioresour Technol.

[CR50] Yoon SH, Ha SM, Kwon S, Lim J, Kim Y, Seo H, Chun J (2017). Introducing EzBiocloud: a taxonomically united database of 16s rRNA gene sequences and whole-genome assemblies. Int J Syst Evol Microbiol.

[CR51] Yuan Y, Liu Y, Li BK, Wang B, Wang SY, Peng YZ (2016). Short-chain fatty acids production and microbial community in sludge alkaline fermentation: long-term effect of temperature. Bioresour Technol.

[CR52] Zhang SS, Yang W, Chen H, Liu B, Lin BX, Tao Y (2019). Metabolic engineering for efficient supply of acetyl-coa from different carbon sources in *Escherichia coli*. Microb Cell Fact.

[CR53] Zhang X-H, Ahmad W, Zhu XY, Chen JX, Austin B (2021). Viable but nonculturable bacteria and their resuscitation: implications for cultivating uncultured marine microorganisms. Mar Life Sci Technol.

