# Supplementary material for: Short-chain fatty acids (SCFAs) as potential resuscitation factors that promote the isolation and culture of uncultured bacteria in marine sediments
Source: Mar Life Sci Technol. 2023 Jul 29;5(3):400–14. doi: 10.1007/s42995-023-00187-w (PMC10449756; doi:10.1007/s42995-023-00187-w)
Supplement: Supplementary file 1 — Supplementary file1 (DOCX 3088 KB) [file 42995_2023_187_MOESM1_ESM.docx]

Short-chain fatty acids (SCFAs) as potential resuscitation factors that promote the isolation and culture of uncultured bacteria in marine sediments

Chun-Shui Sun^1,2,3^, Liu-Yan Zhou^4^, Qi-Yun Liang^1^, Xiao-Man Wang^5^, Yi-Xuan Lei^1^, Zhen-Xing Xu^6^, Feng-Qing Wang^7^, Guan-Jun Chen^1,2^, Zong-Jun Du^1,2,3 *^, Da-Shuai Mu^1,2,3*^

^1^ Marine College, Shandong University, Weihai 264209, China

^2^ State Key Laboratory of Microbial Technology, Institute of Microbial Technology, Shandong University, Qingdao 266237, China

^3^ Weihai Research Institute of Industrial Technology of Shandon University, Weihai 264209, China

^4^ Institute of Microbiology Applications, Xinjiang Academy of Agricultural Sciences, Urumqi 830000, China

^5^ Tancheng County Inspection and Testing Center, Tancheng 276100, China

^6^Department of Applied Biological Chemistry, Graduate School of Agricultural and Life Sciences, The University of Tokyo, Bunkyo-ku, Tokyo, 113-8657 Japan

^7^Max Planck Institute for Marine Microbiology, Celsiusstraße 1, 28359 Bremen, Germany

*Address correspondence to:

Da-Shuai Mu, E-mail: dashuai.mu@sdu.edu.cn;

Zong-Jun Du, E-mail: duzongjun@sdu.edu.cn.


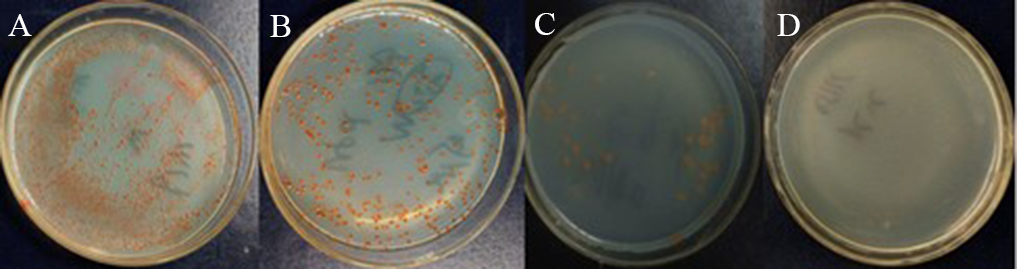


**Supplementary Fig. S1 Colony morphology of *Marinilabilias salmonicolor* when it was stored at 4℃ for (A)0d, (B)15d, (C)60d and(d) 98d.**


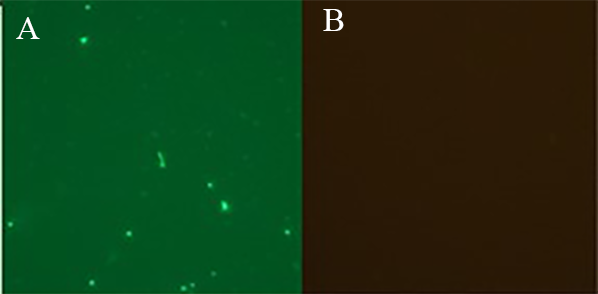


**Supplementary Fig. S2 Live(A) and dead(B) cell micrographs of *Marinilabilias salmonicolor***
